# Effects of walnut shell biochar feed additive on rumen fermentation, nutrient utilization, and performance in fattening lambs

**DOI:** 10.1016/j.vas.2025.100515

**Published:** 2025-09-29

**Authors:** Mehri Montazerharzand, Hamid Paya, Akbar Taghizadeh, Ali Hosseinkhani, Mohammad Ramin

**Affiliations:** a Department of Animal Science, Agricultural Faculty, University of Tabriz, Tabriz, Iran; b Department of Applied Animal Science and Welfare, Swedish University of Agricultural Sciences, 90183, Umeå, Sweden

**Keywords:** Biochar, Fattening lambs, Rumen fermentation, Digestibility

## Abstract

Converting agricultural waste into biochar offers a promising approach to improve animal nutrition and mitigate environmental impacts. This study investigated the effects of dietary supplementation with walnut shell-derived biochar on growth performance, nutrient digestibility, and rumen fermentation characteristics in fattening male lambs. Twelve lambs (initial body weight: 34.4 kg) were randomly assigned to two dietary treatments: a control diet and a diet supplemented with 1 % walnut shell biochar, over a period of 60 days. The inclusion of 1 % walnut shell biochar did not significantly affect dry matter intake (DMI) (*p* = 0.08), average daily gain (ADG) (*p* = 0.06), or feed conversion ratio (FCR) (*p* = 0.47). However, lambs fed the walnut shell biochar diet had a higher final body weight compared to the control group (*p* = 0.05). In contrast, lambs fed the walnut shell biochar -supplemented diet showed significantly higher digestibility coefficients of organic matter (OM), neutral detergent fiber (NDF), and acid detergent fiber (ADF) (*p* < 0.05). Rumen fermentation parameters were also influenced by the walnut shell biochar supplement, with increased total volatile fatty acid (VFA) concentration (*p* = 0.03), higher propionate levels (*p* = 0.054), and reduced protozoa counts (*p* = 0.0003), while rumen pH (*p* = 0.76) and ammonia nitrogen (N—NH3) concentrations (*p* = 0.64) remained unaffected. These findings suggest that walnut shell biochar may improve fiber and organic matter digestibility and beneficially modulate rumen fermentation without compromising growth performance in lambs.

## Graphical abstract

**Figure:**
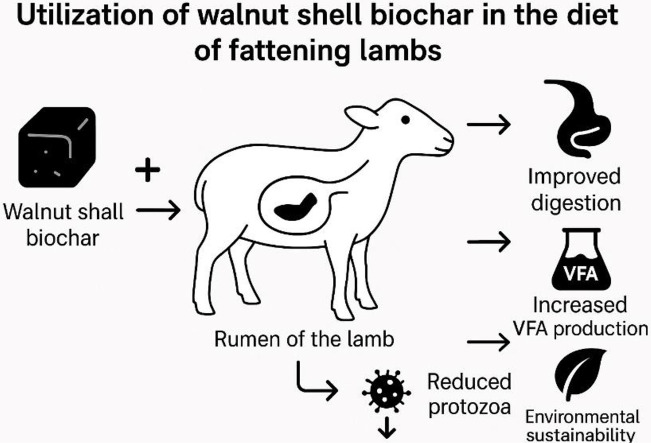

## 1 Introduction

Iran is one of the major centers of walnut (Juglans regia) production in the world, where this tree species has a long history of cultivation and utilization (Hassani et al., 2020). Walnuts are the second most important nut crop after pistachios in terms of production and rank third in cultivated area, covering about 5 % of Iran’s orchards (Hassani et al., 2020). According to the Food and Agriculture Organization (FAO, 2017), Iran accounts for approximately 9.1 % of global walnut production, making it the third-largest producer, and ranks fifth globally in terms of orchard area (4.9 %).

The hard shell of the walnut, generated during processing, is frequently discarded or burned. This practice contributes to landfill waste and creates environmental hazards (McNeill et al., 2024). Converting agricultural residues into valuable products, including compost, animal feed, fertilizers, bioenergy, fibers and biomaterials, and biochar, is considered an environmentally effective strategy (McNeill et al., 2024; Thu & Loan, 2024; Hoang et al., 2024; Phiri et al., 2024). Among such options, biochar production from biomass residues has attracted attention due to its simplicity, low cost, and minimal infrastructure requirements (Awogbemi & Von Kallon., 2023a). Key reasons for this include the simplicity of the production process, easy access to diverse and inexpensive raw materials, and positive environmental impacts like lowering greenhouse gas emissions, improving soil quality, and adsorbing toxins and heavy metals (Awogbemi & Von Kallon, 2023b).

Biochar is a carbon-rich, porous, and amorphous solid derived from thermal decomposition of organic materials under limited oxygen conditions (Rabbani et al., 2024). It has gained interest not only for soil amendment and carbon sequestration, but also for its potential role in circular bioeconomy and livestock nutrition (Reggi et al., 2024). Its high surface area and functional groups enable it to interact with gut microbiota, bind toxins, and potentially modulate ruminal fermentation.

Rumen microorganisms play an important role in the breakdown and conversion of feed materials into energy and nutrients; however, some of these microbes can reduce feed efficiency and cause the loss of certain nutrients (Patra et al., 2009). To enhance microbial efficiency and improve overall animal performance, the use of feed additives has gained increasing attention (Kumar et al., 2022). These additives have been shown to improve animal productivity (Hegazy et al., 2023; Wahid et al., 2024), enhance microbial efficiency (Brenda et al., 2024), and serve as potential alternatives to antibiotics (Hussain et al., 2024). Biochar is one such feed additive that has been investigated for these purposes.

Although several *in vitro* studies have demonstrated promising effects of biochar on rumen fermentation and gas production (Qomariyah et al., 2023; Van Dung et al., 2024; Tamayao et al., 2021), *in vivo* evidence remains limited. For example, Leng et al. (2012) reported improved growth and feed efficiency in cattle supplemented with rice husk biochar (0.6 % of dietary DM), while Keba et al. (2023) reported increased feed intake and digestibility in rams receiving corn stalk biochar. Positive effects on animal performance have also been documented in goats and lambs (Khoa et al., 2018; Hien et al., 2018; Saroeun et al., 2018). However, some studies reported neutral effects; Lind et al. (2024) found no impact of biochar on daily weight gain in lambs, and Al-Kindi et al. (2017) observed no changes in feed intake or rumen microbial communities in goats supplemented with coconut shell biochar. Ni et al. (2024) also reported minimal effects of standard or enriched biochar on rumen fermentation and microbial populations in Holstein steers.

Such inconsistencies may be attributed to differences in biochar source, production conditions, dosage, and animal species. Indeed, the physicochemical properties of biochar are greatly influenced by the feedstock type and pyrolysis parameters; for example, pyrolysis temperature affects the elemental composition, pore structure, surface area, and functional groups of biochar (Tripathi et al., 2016).

Although biochar has demonstrated positive effects on rumen microbial activity and animal performance, attention must also be given to its potential inherent contaminants. The inherent contaminants of biochar originate from its raw materials and may include heavy metals (such as lead, chromium, and cadmium) or organic pollutants, such as polycyclic aromatic hydrocarbons (PAHs). During pyrolysis, some of the heavy metals may volatilize, while other compounds remain in the biochar. These contaminants can be released into the environment during production and application, potentially posing environmental and health risks (Dong et al., 2025).

Despite the growing interest, most current biochar research is focused on its role in soil and environmental remediation, with relatively limited attention to its potential as a dietary supplement for livestock. Therefore, the present study was conducted to evaluate the effect of dietary supplementation with biochar derived from walnut shell waste on rumen fermentation, nutrient digestibility, and growth performance in fattening lambs. This research contributes to the emerging field of animal feeding strategies through valorization of agricultural residues.

## 2 Materials and methods

### 2.1 Ethical statement

The methods used in this study were approved by the Biomedical Ethics Committee of Tabriz University (IR.TABRIZU.REC.1403.129).

### 2.2 Sample preparation and biochar production

Walnut shell waste was collected and washed with water to remove external impurities, then dried at room temperature for 3 days. To produce biochar, the sample was ground using a grinder with a 5 mm sieve, then placed in an electric furnace under argon gas (5 L per minute) at a temperature of 500 °C with a heating rate of 5 °C per minute for 4 h. After the furnace temperature gradually returned to room temperature, the biochar was removed from the furnace and stored in sealed plastic containers at room temperature. The mass yield of the walnut shell biochar was calculated as the percentage of the biochar produced relative to the initial weight of the raw material. The ash content was measured by burning the biochar at 750 °C for 6 h (ASTM, 2007).

The elemental composition (carbon, nitrogen, hydrogen, and sulfur (CHNS)) of the walnut shell biochar was analyzed using an elemental analyzer (Costech-ECS 4010-USA). The oxygen content of the samples were calculated by subtracting the sum of carbon, nitrogen, hydrogen, sulfur, and ash from 100 (Table 1).

**Table 1:** Characteristics of biochar made from walnut shell.

mass yield (%)	32.2
Ash content (%)	2.42
Element analysis (wt%)	
Carbon	84.5
Hydrogen	1.30
Nitrogen	1.25
Sulphur	0.23
Oxygen	10.3

### 2.3 Animal preparation, diets, and experimental treatments

This study was conducted on 12 male Qezel lambs (2 to 3 months old) with an average weight of 34.5 kg, following a completely randomized design (6 animals/treatment). The experimental treatments included: 1) control diet without biochar, and 2) control diet with 1 % walnut shell biochar (based on dry matter intake (DMI) percentage, applied as a top-dressing). The experimental diet was formulated according to the NRC (2007) recommendations to meet the nutritional requirements of lambs (Table 2). The lambs were fed twice daily (8 AM and 6 PM) with a total mixed ration (TMR) consisting of 40 % forage and 60 % concentrate. Before the start of the trial, all animals were vaccinated against internal and external parasites and vaccinated for enterotoxemia. The lambs were housed individually, with free access to water and feed.

**Table 2:** Ingredients and chemical composition of experimental diets (% DM).

Ingredients (%)		Chemical composition (%)	
Alfalfa	30.0	Dry matter	95.3
Wheat straw	10.0	Crude protein	16.8
Barley grain	30.0	Ash	7.68
Wheat bran	20.7	Ether extracts	1.65
Soybean meal	7.80	Neutral detergent fiber	38.1
Salt	0.50	Acid detergent fiber	20.0
Sodium bicarbonate	0.50	Metabolizable energy (Mcal/kg)	2.01
Minerals and vitamins supplement	0.50		

The study included a 14-day acclimatization period and a 60-day experimental period during which changes in body weight (BW) and DMI were recorded. Throughout the experimental period, daily feed intake was weighed, and feed was provided ad libitum. The remaining feed was measured daily, and the feed intake of each animal was calculated by subtracting the remaining feed from the total distributed feed. The lambs were weighed biweekly before the morning feed.

### 2.4 Chemical composition and apparent digestibility determination

To determine the chemical composition and apparent digestibility, feed and fecal samples (*n* = 6) were collected during the last 5 days of the experimental period. Daily feed samples were obtained from the TMR provided to each lamb. Fecal samples were collected directly from the rectum of each lamb 3 h after morning feeding. The daily samples were then composited separately to generate one representative feed sample and one representative fecal sample per lamb for chemical analysis. The samples were dried at 60 °C for 48 h and ground using a 2-mm sieve. The dry matter, ash, crude fat, and crude protein were measured according to the methods outlined by AOAC (2005), and acid detergent fiber (ADF) and neutral detergent fiber (NDF) were determined according to the Van Soest et al. (1991) method, without using heat-stable alpha-amylase and sodium sulfite. The chemical composition of the feed is reported in Table 2.

Apparent digestibility was calculated using acid-insoluble ash (AIA) as an internal marker (Van Keulen & Young, 1977), according to the following formulas:

$$\begin{array}{cc} \text{Dry}\;\text{Matter}\;\text{Digestibility}\;\left(\%\right) & =100\\ & -\left[\left(\%\mathrm{AIA}\;\mathrm{in}\;\mathrm{feed}\;/\;\%\mathrm{AIA}\;\mathrm{in}\;\mathrm{feces}\right)\times 100\right] \end{array}$$

$$\begin{array}{cc} \text{Nutrient}\;\text{Digestibility}\;\left(\%\right) & =100-[\left(\%\mathrm{AIA}\;\mathrm{in}\;\mathrm{feed}\;/\;\%\mathrm{AIA}\;\mathrm{in}\;\mathrm{feces}\right)\\ & \times \left(\%\mathrm{Nutr}\mathrm{ient}\;\mathrm{in}\;\mathrm{feces}\;/\;\%\mathrm{Nutr}\mathrm{ient}\;\mathrm{in}\;\mathrm{feed}\right)\times 100] \end{array}$$

### 2.5 Sampling and analysis of rumen fluid

To evaluate the effect of walnut shell biochar supplementation on rumen fermentation parameters (*n* = 3) such as pH, ammonia nitrogen (N—NH_3_), volatile fatty acids (VFAs), and protozoa, rumen fluid samples were collected from the lambs on the last day of the experimental period, 2 h after the morning feed, using an esophageal tube and filtered with a 4-layer cloth. Immediately after sampling, pH was measured using a portable pH meter.

For protozoa counting, the collected rumen fluid was mixed with a formalin solution (100 mL of 37 % formaldehyde and 8.5 g of Merck salt diluted to 1 L with distilled water) at a ratio of 1:4 (1 part rumen fluid to 4 parts formalin) to stop microbial activity and was stored in the refrigerator until counting. During counting, a drop of each sample was placed on a slide, covered with a coverslip, and protozoa were counted under a light microscope at 10x magnification in the four-cornered cells (Ivan et al., 2013).

To measure N—NH_3_, the rumen fluid was mixed with 0.2 N hydrochloric acid at a 5:1 ratio (rumen fluid : HCl), and to measure VFA, it was mixed with 50 % sulfuric acid at a 1:50 ratio (1 part fluid to 50 parts H_2_SO_4_). The samples were stored at -20 °C until analysis. N—NH_3_ was measured using a spectrophotometer (wavelength 630 nm) following the method of Broderick and Kang (1980). VFAs were measured using gas chromatography (Agilent, 6890 N, USA; Animal Science Research Institute of Iran). The injector and detector temperatures were set at 240 °C and 270 °C, respectively. The nitrogen flow rate (carrier gas) was 1.8 mL/min, and the detector was an FID. The initial column temperature (DB-FFAP capillary column, J&W 123–3232, 30 m length, 320 µm inner diameter, 0.25 µm film thickness) was set at 80 °C, held for 2 min, and then increased at 10 °C/min until reaching 160 °C. A 1 µL sample volume was injected with a split ratio of 1:20, and the total run time was 20 min. 2-ethyl butyric acid was used as an internal standard. Calibration standards were prepared using pure organic acid standards (Merck, Germany) at concentrations ranging from 0.05 to 100 mM.

### 2.6 Statistical analysis

The experiment was conducted in a completely randomized design. Non-repeated data were analyzed using the Analysis of Variance (ANOVA) model in SAS software (version 2.9) with the GLM procedure. To compare means, Duncan's multiple range test was used at a significance level of 0.05 (Model 1). Repeated measures data (feed intake and lamb performance) were analyzed using the Mixed procedure in SAS software. The least square means were compared using the Tukey-Kramer test at the 0.05 level of significance (Model 2).

Model1:Yij=μ+Ti+eij

Model2:Yij=μ+Ti+Pj+β(Xi−X)+eij

In these models, Yij represents the value of each observation, Ti represents the treatment effect, μ is the overall mean, Pj represents the period effect, β(Xi - X) is the covariate effect (initial weight), and eij is the experimental error. It is important to note that to adjust for autocorrelation observed in the measured data over time, covariance structures for model fitting were evaluated based on the Akaike Information Criterion (AIC), where the smallest value indicates the best model (Table 3).

**Table 3:** AIC values (Akaike information criterion) for each model.

	AIC						
Parameters	Default	CS	AR(1)	TOEP	UN	ANTE(1)	ARH(1)
BW	173	165	156	161	162	164	159
ADG	457	457	458	461	455	461	463
DMI	545	537	532	534	541	537	534
FCR	112	113	114	116	116	114	118

BW = body weight; ADG= average daily gain; DMI= dry matter intake; FCR= feed conversion ratio.

CS: Compound symmetry, AR(1): Autoregressive(1), Toep: Toeplitz, UN: Unstructured, ANTE(1): Antedependence, ARH(1): Heterogeneous AR(1).

## 3 Results

### 3.1 Feed intake and growth performance of lambs

**Fig. 1:**
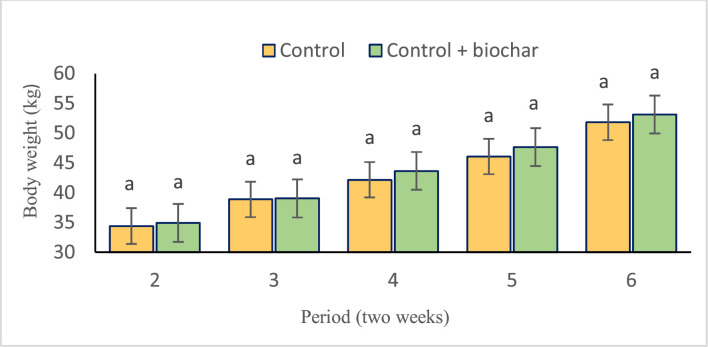
Comparison of the effect of period (time) on body weight of fattening lambs in experimental treatments (control and control+biochar).

The effect of walnut shell biochar supplementation on feed intake and growth performance of fattening lambs is presented in Table 4. According to Table 4, there was no significant difference in the initial body weight between the control group and the 1 % walnut shell biochar-supplemented group (*p* = 0.22). However, the effect of walnut shell biochar on final body weight was statistically significant, with the highest body weight observed in lambs fed with walnut shell biochar (*p* = 0.05). Despite this, no significant interaction was observed between treatment and time regarding body weight over the experimental period (Fig. 1) (*p* > 0.05). There were no significant differences among treatments in terms of average DMI (*p* = 0.08), feed conversion ratio (FCR) (*p* = 0.47), and average daily gain (ADG) (*p* = 0.06).

**Table 4:** Effect of experimental treatments on growth performance of fattening lambs.

	Experimental treatments^1^			*p*-value		
Parameters	Con	Con+WS-B	SEM	Treatment	Period	Treatment × Period
Initial BW (kg)	34.6	34.3	0.88	0.22	–	–
Final BW (kg)	42.6^b^	43.6^a^	0.32	0.05	<0.0001	0.24
ADG (g/d)	311	337	8.92	0.06	0.0008	0.3
DMI (g/d)	1663	1810	55.3	0.08	<0.0001	0.36
FCR	5.43	5.23	0.18	0.47	0.14	0.22

1 Experimental diets contained without biochar as a control (CON) and walnut shell biochar (WS-B 1 %).

a,b Values within a row with different superscripts differ significantly at *p* < 0.05. BW= body weight; ADG = average daily gain; DMI = DM intake; FCR = feed conversion ratio.

### 3.2 Dry matter and nutrient digestibility

According to the results presented in Table 5, the digestibility of organic matter (OM) and fibrous nutrients (NDF and ADF) was significantly higher in lambs fed with 1 % walnut shell biochar compared to those fed the control diet (*p* < 0.05).

**Table 5:** Effect of experimental treatments on apparent digestibility of nutrients.

	Experimental treatments^1^			
Nutrients (%)	Con	Con+WS-B	SEM	*p*-value
Dry matter	71.8	73.1	0.97	0.38
Organic matter	73.0^b^	75.2^a^	0.73	0.04
Crude protein	73.1	75	0.96	0.23
Neutral detergent fiber	63.3^b^	66.0^a^	0.8	0.04
Acid detergent fiber	62.4^b^	65.0^a^	0.67	0.02

1 Experimental diets contained without biochar as a control (CON) and walnut shell biochar (WS-B 1 %).

a,b Values within a row with different superscripts differ significantly at *p* < 0.05.

### 3.3 Rumen fermentation parameters

The effects of 1 % walnut shell biochar supplementation on rumen fermentation parameters are presented in Table 6. According to the results, walnut shell biochar significantly influenced protozoa counts, total VFAs, and branched-chain VFAs (specifically isobutyrate) (*p* < 0.05), whereas ruminal N—NH_3_ (*p* = 0.64) and pH (*p* = 0.76) were not significantly affected.

**Table 6:** Effect of experimental treatments on rumen fermentation parameters.

	Experimental treatments*			
Parameters	Con	Con+WS-B	SEM	*p*-value
pH	6.2	6.2	0.07	0.76
Protozoa (*n* × 10^5^/ml)	13.1^a^	11.1^b^	0.12	0.0003
N-NH_3_ (mM)	15.8	15	1.13	0.64
VFA (mM)				
Acetate	86.5^b^	118^a^	7.33	0.03
Propionate	28.1	42.5	3.8	0.054
Butyrate	12.0^b^	18.2^a^	0.37	0.0003
Iso-Butyrate	0.64^b^	0.92^a^	0.02	0.0005
Valerate	2.22	3	0.21	0.09
Iso-Valerate	0.8	1.06	0.07	0.052
Total VFA	130^b^	186^a^	12.1	0.03
Acetate/propionate	3.08	2.66	0.2	0.2

⁎ Experimental diets contained without biochar as a control (CON) and walnut shell biochar (WS-B 1 %).

a,b Values within a row with different superscripts differ significantly at *p* < 0.05.

## 4 Discussion

### 4.1 Feed intake and growth performance of lambs

The present results contribute to a better understanding of the effects of biochar under *in vivo* conditions, where so far limited evidence has been reported.

In the present study, the absence of a significant effect of 1 % walnut shell biochar on DMI suggests that inclusion of this additive is unlikely to disrupt palatability or nutrient supply to the animal. Although the direct effect of walnut shell biochar on rumen microbes was not assessed in this study, the observed increase in nutrient digestibility in lambs fed biochar (Table 5) indicates that this additive may have selectively enhanced the activity of ruminal fibrolytic bacteria. Moreover, since increased production of VFAs, particularly acetate, in the rumen is a key indicator of enhanced fiber digestibility in ruminants, the final weight observed in lambs fed walnut shell biochar can be attributed to both higher VFA production (Table 6) and improved nutrient digestibility. Biochar’s porous structure has been proposed to provide a favorable microbial habitat for fibrolytic bacteria, thereby enhancing their fermentative activity and fiber degradation (Kumar et al., 1987).

The findings of the present study are consistent with those of previous researchers. For instance, Mirheidari et al. (2019) reported no change in DMI in dairy sheep fed biochar derived from walnut shells and poultry litter compared to controls. Similarly, Benhissi et al. (2025) observed no effect on DMI when biochar was combined with probiotics in dairy sheep diets. In the study by Keba et al. (2023), corn cob biochar supplementation at levels of 1.5, 3, and 4.5 g/day was evaluated for its effects on growth performance and carcass characteristics of sheep. The corn cob biochar supplementation significantly improved the final weight and ADG of the experimental sheep (*p* < 0.001). However, the treatment with 1.5 g/day biochar resulted in the highest final weight and ADG among all groups. FCR was significantly reduced by the addition of corn cob biochar (*p* < 0.001), although there was no significant difference in FCR improvement among the different supplementation levels. Corn cob biochar supplementation also significantly enhanced carcass percentage, rib weight, and brisket weight (*p* < 0.001). The study observed significant effects of the treatments on the weights of internal organs; however, all values remained within the normal range for sheep, and no damage to any organs was reported. Therefore, the study demonstrated that inclusion of corn cob biochar in sheep diets improves fattening performance and carcass traits without any adverse effects on animal health. The present study also aligns with previous research by Silivong and Preston (2015) and Leng et al. (2012), who reported increased body weight in calves and goats, respectively.

### 4.2 Dry matter and nutrient digestibility

The inclusion of biochar in the diet may support the creation of new microbial habitats within the rumen, increasing the surface area for biofilm formation and enhancing the interaction between various microbial populations. This, in turn, may improve bacterial adhesion to feed particles, facilitating more effective digestion (Leng, 2014). However, as no microbial analysis was performed in this study, the proposed mechanisms should be interpreted with caution. In the present study, the findings on digestibility indicate a positive effect of 1 % walnut shell biochar supplementation on nutrient utilization in lambs. Similarly, Mirheidari et al. (2020) reported the highest digestibility coefficients of DM, OM, CP, and NDF in lambs supplemented with 1 % walnut shell biochar compared to the control group, indicating that biochar may stimulate microbial communities and enhance ruminal fermentation.

In a study by Winders et al. (2019), OM and NDF digestibility improved during the growing phase in cattle fed biochar, although OM digestibility showed a linear decline during the finishing phase, and NDF digestibility was not affected. However, in a study by Silivong and Preston (2015), the digestibility of DM, OM, and CP in goats fed Bauhinia-based diets was not influenced by biochar supplementation. Similarly, Terler et al. (2023) found that neither biochar nor the combination of biochar and urea had a significant effect on nutrient digestibility, indicating that dietary effects may vary depending on species, diet composition, and biochar type.

### 4.3 Rumen fermentation parameters

The normal ruminal pH range for sheep is between 6.4 and 6.8. Values below 5.5 or above 7 are considered abnormal (Jasmin et al., 2011). The average pH values observed in this study fell within the normal range, suggesting that fermentation was proceeding efficiently and was not disrupted by microbial activity. Although the difference in N—NH_3_ concentrations was not statistically significant (*p* = 0.64), the numerical reduction observed may reflect increased utilization of ammonia by cellulolytic bacteria, which rely on ammonia as their sole nitrogen source (Bryant & Robinson, 1961). Additionally, it is well known that rumen protozoa enhance dietary protein degradation and rapidly release NH_3_. They also recycle nitrogen by engulfing and digesting bacteria. Indeed, NH_3_ concentration in rumen fluid is positively correlated with protozoa populations, and animals that are defaunated typically exhibit lower NH_3_ levels (Leng & Nolan, 1984). In the current study, the reduction in protozoa was aligned with a decrease in N—NH_3_ concentration. The observed decrease in protozoa along with the reduction in N—NH₃ may indicate changes in nitrogen metabolism. However, in the absence of data on microbial protein, this cannot be considered a definitive indicator of improved nitrogen efficiency. However, other studies (Leng et al., 2012; Garillo et al., 1995) reported increased NH_3_ concentrations as a result of biochar or activated carbon supplementation in cattle and sheep.

The increase in total VFA concentration in the present study may be attributed to improved OM degradation. Specifically, supplementation with 1 % walnut shell biochar led to a rise in ruminal propionate levels. This aligns with previous research suggesting that biochar may influence ruminal microbial diversity and potentially support propionate-producing bacterial populations (Martinez-Fernandez et al., 2024). However, these mechanisms fall outside the scope of the current study and require further specialized investigation.

Isobutyrate, isovalerate, and valerate are products of branched-chain amino acid fermentation in the rumen. Isovalerate and valerate, in particular, are considered stimulatory compounds that enhance the growth of cellulolytic bacteria (Guo et al., 2021). Therefore, biochar addition may support the proliferation of certain cellulolytic bacterial populations in the rumen, ultimately improving acetate production and NDF digestibility. The observed increase in NDF disappearance indicates that biochar may play an effective role in supporting microbial population growth (Saleem et al., 2018). In a study by Pereira et al. (2014), total VFA and acetate concentrations increased with biochar supplementation, while propionate levels decreased and butyrate remained unaffected. Conversely, other studies (McFarlane et al., 2017; Mirheidari et al., 2020; Ni et al., 2024) found that VFAs were not significantly affected by biochar supplementation.

## 5 Conclusions

The present study focused on biochar produced from walnut shell waste, highlighting its potential as a value-added feed additive. This approach not only offers a solution for reducing agricultural waste but also contributes to the reutilization of natural resources. Supplementation of walnut shell biochar in the diet of fattening lambs led to improved nutrient digestibility and positively influenced certain rumen fermentation parameters. These findings indicate that biochar has potential as a feed additive in ruminant feeding systems.

## Ethical statement

The methods used in this study were approved by the Biomedical Ethics Committee of Tabriz University (IR.TABRIZU.REC.1403.129).

## CRediT authorship contribution statement

**Mehri Montazerharzand:** Writing – review & editing, Writing – original draft, Methodology, Formal analysis, Data curation. **Hamid Paya:** Writing – review & editing, Writing – original draft, Visualization, Validation, Supervision, Formal analysis, Data curation, Conceptualization. **Akbar Taghizadeh:** Writing – review & editing, Writing – original draft, Investigation, Conceptualization. **Ali Hosseinkhani:** Writing – review & editing, Writing – original draft, Methodology, Conceptualization. **Mohammad Ramin:** Writing – review & editing, Validation, Supervision, Methodology, Conceptualization.

## Declaration of competing interest

The authors declare that they have no known competing financial interests or personal relationships that could have appeared to influence the work reported in this paper.
